# Delayed Chronotropic Response due to Autonomic Imbalance Presenting as Dyspnea on Early Exertion: A Complication of Bariatric Surgery

**DOI:** 10.7759/cureus.3212

**Published:** 2018-08-27

**Authors:** Shazib Sagheer, Abu Baker Sheikh, Mohammed Alkubeysi, Paul Andre, Christopher Bunn, Shmuel Inbar

**Affiliations:** 1 Internal Medicine, University of New Mexico School of Medicine, Albuquerque, USA; 2 Internal Medicine, University of New Mexico, Albuquerque, USA; 3 Internal Medicine, University of New Mexico Hospital, Albuquerque , USA; 4 Internal Medicine, University of New Mexico Hospital, Albuquerque, USA

**Keywords:** chronotropic response, bariatric surgery, dyspnea, autonomic imbalance

## Abstract

Bariatric surgery has shown promising outcomes in improving overall morbidity and mortality in morbidly obese patients. Cardiovascular risk reduction from weight loss is well known in the literature. However, little is highlighted about the cardiovascular complications of massive and rapid weight loss associated with bariatric surgery. These complications result mainly from autonomic imbalance manifesting as increased parasympathetic tone and a decrease in sympathetic response. This imbalance is a consequence of hormonal changes associated with massive weight loss. We present a unique case which is a demonstration of the aforementioned changes. Our patient presented with dyspnea during an early phase of exercise with the resolution of symptoms with the continuation of exercise.

## Introduction

Due to rising prevalence of obesity in the world, bariatric surgery is gaining more importance. According to the American Society for Metabolic and Bariatric Surgery (ASMBS) 196,000 bariatric surgeries were performed in the USA in 2015 [1]. Cardiovascular risk reduction after weight loss is well documented in literature. Little is mentioned about the impact of weight loss on symptomatic bradycardia. Our case is a unique presentation of autonomic imbalance causing symptomatic bradycardia after massive weight loss associated with Roux-en-Y gastric bypass surgery.

## Case presentation

A 53-years-old female with the history of morbid obesity status post-Roux-en-Y gastric bypass surgery presented with a chief complaint of dyspnea on exertion and intermittent substernal chest pain. The patient reported that for the past two months she would feel very short of breath during early 10-15 minutes of exercise, however, with continued exertion her symptoms resolved. The patient then began to develop intermittent substernal chest pain, not associated with exercise, which prompted her to present to the emergency department. On further history, the patient stated that she has had dyspnea on early exercise after massive weight loss since bariatric surgery, but her symptoms worsened after she moved to high altitude in Albuquerque two months ago. The patient had undergone bariatric procedure five years prior to presentation and subsequently lost 100 pounds with an 18-point drop in body mass index (BMI).

On emergency department visit, the patient’s physical exam revealed resting bradycardia with a heart rate (HR) of 55 beats per minute (BPM) and blood pressure at 89/54. The patient was of normal weight with a BMI of 24. The patient denied any history of tobacco abuse, excessive consumption of alcohol or drug use. She also denied being on any negative ionotropic drugs.

**Figure 1:**
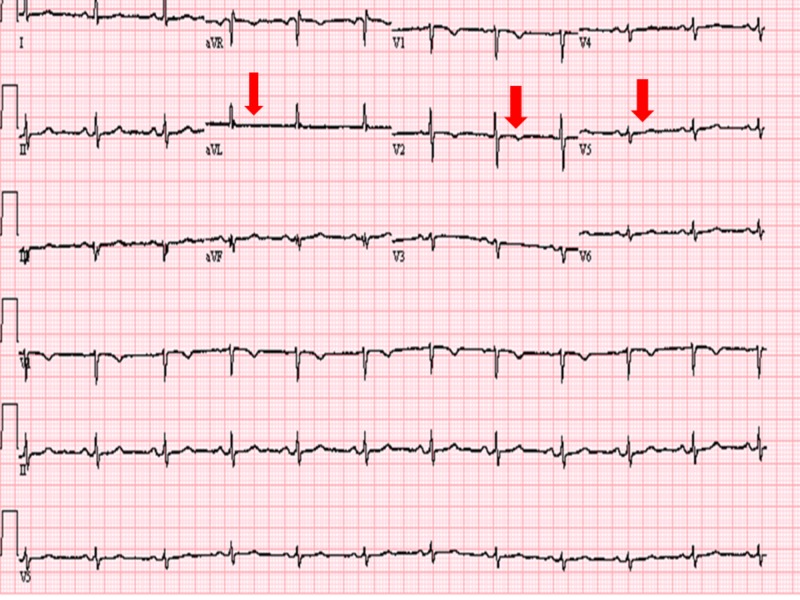
Electrocardiogram showing non-specific ST waves changes (Red arrows).

Electrocardiogram (EKG) revealed non-specific ST waves changes (Figure 1).

High sensitivity troponin I was within normal range (<0.017). Given EKG changes and a strong family history of coronary artery disease (CAD), the patient underwent EKG exercise stress test per the Bruce protocol.

**Figure 2:**
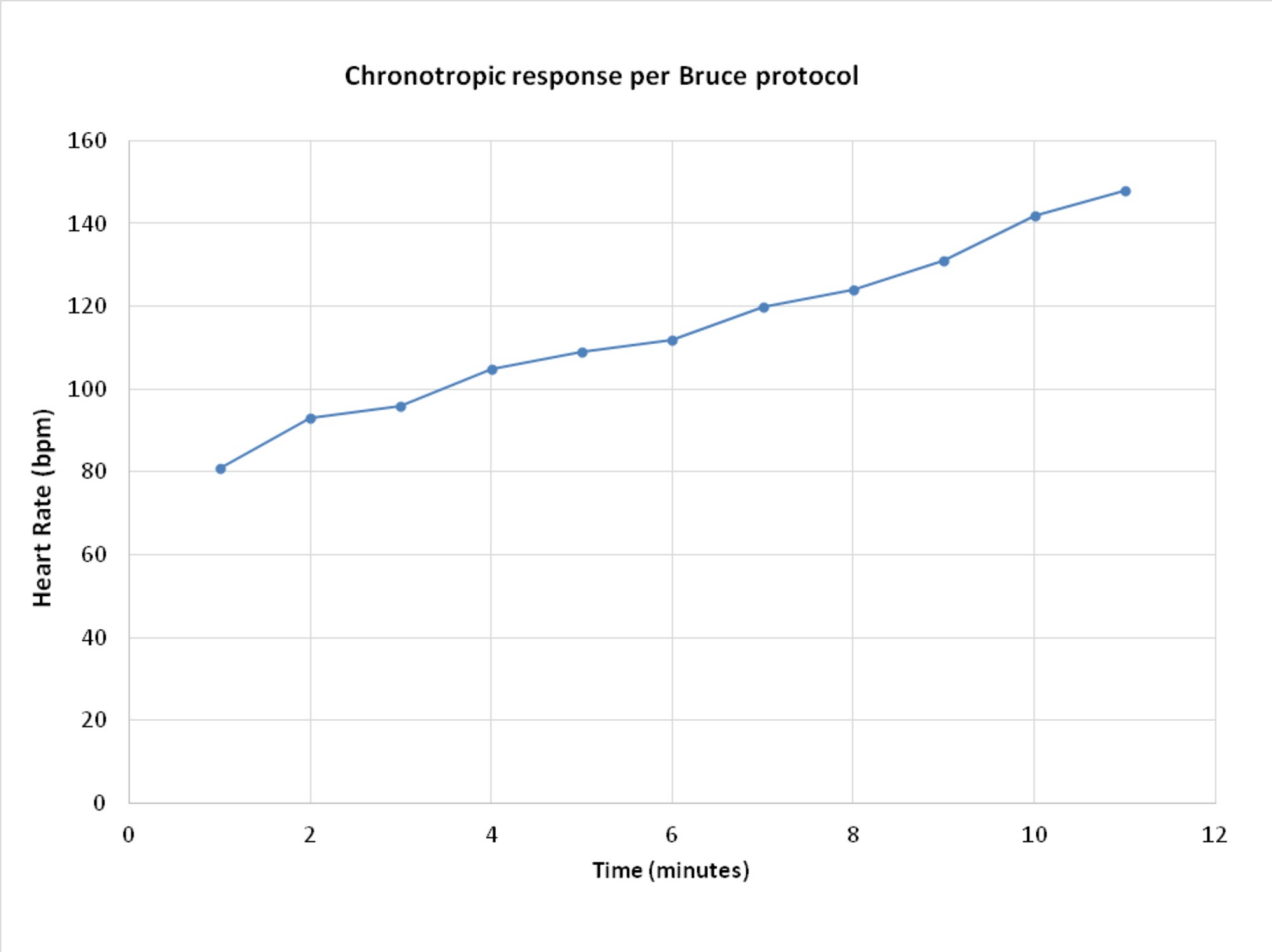
Heart rate (HR) response during exercise stress test per Bruce protocol. HR does not increase substantially until later part of stage III of Bruce protocol. Patient’s symptoms started to resolve at ninth minute of exercise during stage III.

The patient’s resting HR was 68. Stage I of exercise patient’s heart rate was 81. The patient did not experience a significant rise in heart rate until later part of stage III of exercise at 10.4 metabolic equivalents (METs) where her rate increased to 133 beats per minute. The patient did not achieve target HR until stage IV of exercise when her HR did increase to 148 which was 88% of age-predicted HR. Notably, the patient did experience profound dyspnea during the test, which improved once she reached HR of 133 at the ninth minute during stage III of exercise (Table 1) (Figure 2).

**Table 1:** Patient’s heart rate response per Bruce protocol. METs: Metabolic equivalents; MPH: Miles per hour; HR: Heart rate.

Stages	Time (minutes)	HR (bpm)	MPH	Grade	METs
1	1	81	1.7	10	4
	2	93			
	3	96			
2	4	105	2.5	12	7
	5	109			
	6	112			
3	7	120	3.4	14	10
	8	124			
	9	131			
4	10	142	4.2	16	13
	11	148			

**Figure 3:**
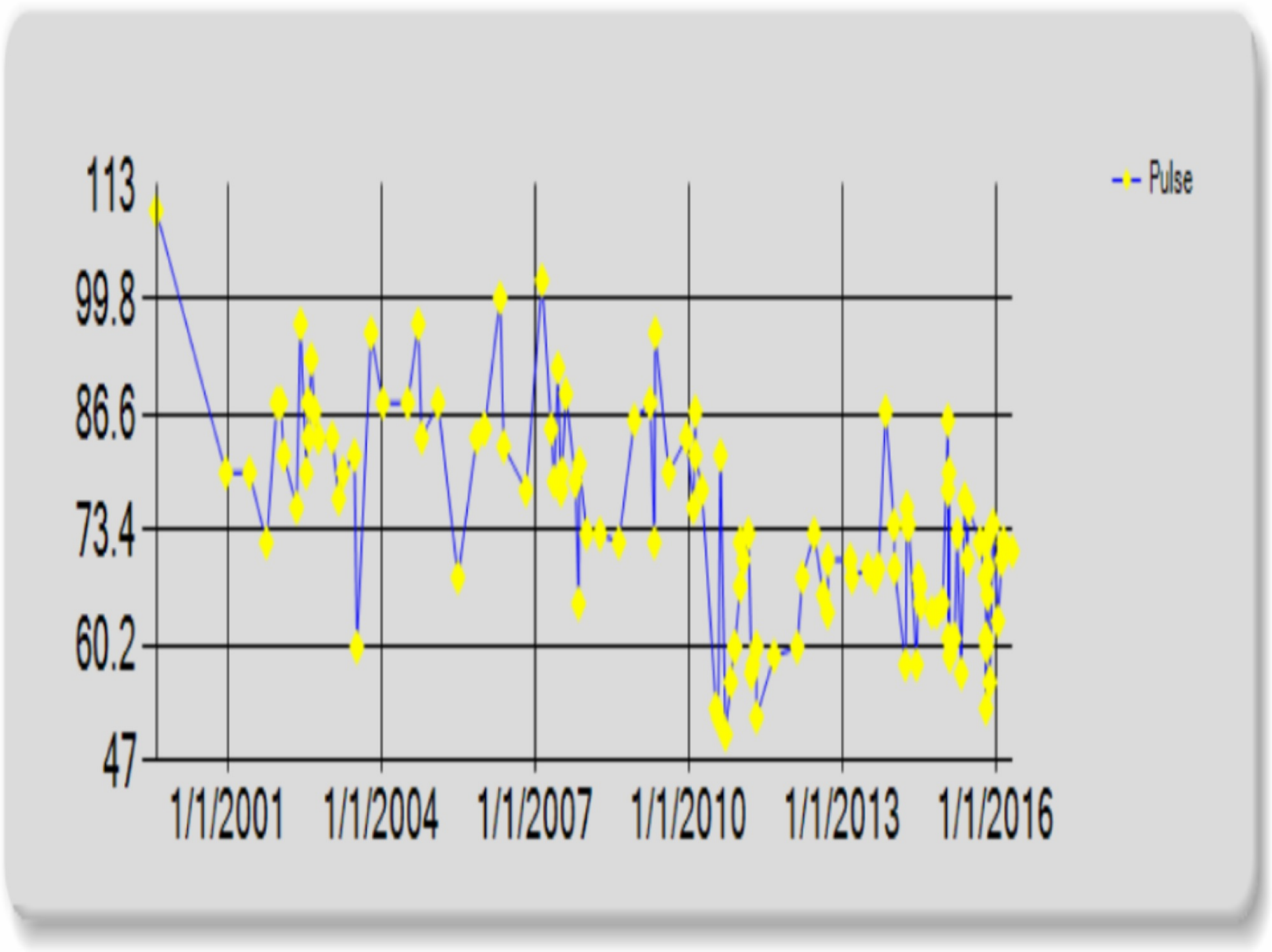
Average heart rate before and after Roux-en-Y surgery.

**Figure 4:**
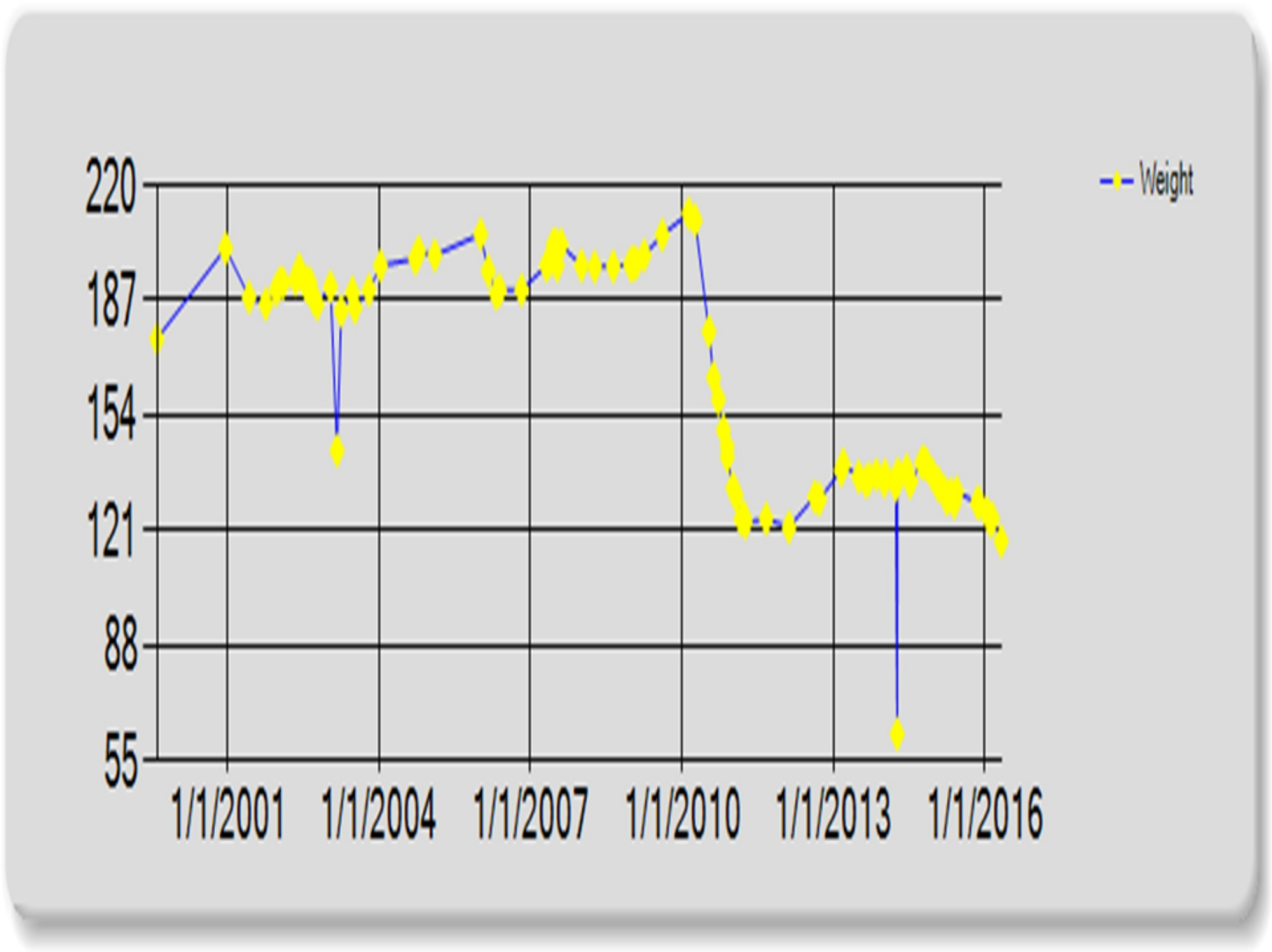
Weight (Kg) before and after surgery.

The patient’s resting bradycardia, hypotension, and delay of appropriate heart rate response during exercise stress testing prompted patient’s chart review. Comparison of resting heart rate before and after significant weight loss revealed a marked difference. Prior to bariatric intervention, the patient’s heart rate averaged in the 80s. After 100 lb weight loss and the decrease in BMI of 46% the patient’s heart rate dropped significantly, averaging in the 50s. The patient’s blood pressure followed a similar trend (Figures 3, 4).

Retrospective chart review also revealed that approximately one year after a Roux-en-Y procedure and 100 pounds of weight loss, the patient presented to her primary care clinician complaining of light-headedness and hypotension at home. This was deemed to be due to “dehydration” related to gastric bypass surgery. Reviewing multiple clinic visits prior to presentation, the patient suffered from a dizzy feeling intermittently for four years prior to presenting to our facility, wherein she had just recently moved to Albuquerque, New Mexico at an elevation of >5,000 feet.

Ultimately, the patient’s chest pain was determined to be due to gastroesophageal reflux disease (GERD) and a large hiatal hernia as a complication of her bariatric intervention. However, her dyspnea on exertion was determined to be secondary to the delayed chronotropic response due to overwhelming cholinergic tone and less sympathetic tone at baseline due to hormonal changes associated with massive weight loss. The patient was counseled about her condition with the plan to follow her symptoms in subsequent clinic visits.

## Discussion

Increased parasympathetic and reduced sympathetic activity after significant weight loss is well documented in the literature. Malik et al. were the first to formally document asymptomatic sinus bradycardia after bariatric surgery-induced weight loss [2]. Analyzing data for 24 months into the post-operative period, they showed an increased incidence of asymptomatic bradycardia in patients who underwent large volume weight loss associated with bariatric surgery. In particular, the incidence of asymptomatic bradycardia increased in relation to greater decreases in BMI.

In addition to reducing blood pressure and decreasing insulin resistance, bariatric surgery weight loss is associated with decrease in left ventricle (LV) mass, improve in LV systolic function and diastolic dysfunction, and overall improved cardiac function due to favorable effects of gut hormones such as glucagon-like peptide-1 (GLP-1) and ghrelin [3]. Massive weight loss comes with significant changes in hormone levels and alterations in autonomic function with the decrease in sympathetic tone and an increase in vagal tone. One study showed increase in sympathetic activity and a decrease in parasympathetic activity with 10% increase in weight gain [2,4].

Seravalle and Grassi have demonstrated sustained and substantial decrease in human skeletal muscle sympathetic nerve activity (SNA) after vertical sleeve gastrectomy-induced weight loss. Sympathetic nerve activity continued to decrease at six and 12 months marks post-procedure. This study also demonstrated a significant decrease in heart rate in the surgically induced weight loss group [5]. In addition to that, several adipokines are associated with heart rate control in an obese population. An elevated level of leptin as seen in obesity has been associated with high heart rate [6].

The molecular mechanism for bradycardia after massive weight loss is not well known. Marked sinus bradycardia in patients with weight loss due to Anorexia Nervosa has been studied. This weight loss is thought to be due to vagal hyperreactivity given its sensitivity to vagolytic drugs. Anorexia Nervosa patients presenting with symptoms, sinus node dysfunction, have been proposed in some studies [7]. Leptin receptors on the plasma membrane of the sinus node, atrial and ventricular myocytes have been found in animal studies [8]. These studies have also demonstrated bradycardia by direct action of leptin on its cardiac receptors and tachycardia by indirect sympathomimetic action. The intracellular signaling pathways of leptin receptors need further study.

Our case is a unique and very rare manifestation of aforementioned changes associated with massive and relatively rapid weight loss. Our patient presented with symptoms in the early phase of exercise with the resolution of symptoms with a continuation of exercise. We believe the patient had an inappropriately higher cholinergic tone at baseline and hence needed higher sympathetic system activation to overcome the cholinergic tone, which could only be achieved with intense exercise. This was manifested as a delayed chronotropic response on stress electrocardiogram. As mentioned above, this imbalance in an autonomic nervous system is due to hormonal changes associated with weight loss.

The patient described above became symptomatic roughly one year post-operatively after weight loss of 100 lbs and a reduction in BMI of 46%. There is no data in the literature to define the incidence of symptomatic sinus bradycardia in this subset of individuals, yet the above case begs that this question is addressed. Similarly, there is a lack of data regarding the best management strategy. In one report, symptomatic bradycardia was treated with an anticholinergic agent with subsequent resolution of symptoms [9].

## Conclusions

In conclusion, while considering other complications of bariatric surgery, sinus bradycardia due to delayed chronotropic response could be helpful in clinical decision making. Especially when symptoms of dyspnea, fatigue and exercise intolerance can easily be associated with a nutritional deficiency after weight loss. By explaining the aforementioned symptoms as a manifestation of autonomic imbalance associated with massive weight loss in bariatric surgery, we can avoid expensively time-consuming, and potentially invasive investigations and manage symptoms conservatively. The incidence of symptomatic bradycardia and the best management strategy needs further studies.
